# The Endowment of Pathological Research

**Published:** 1908-05-30

**Authors:** 


### The Hospital

A Journal of

The Medical Sciences and Hospital Administration.

New Series. No. 63, Vol. III. [No. 1135, Vol. XLIV.] SATURDAY, MAY 30, 1908.

### The Endowment of Pathological Research.

The high standard of altruism which our profession justly boasts as one of its most valued possessions has undoubtedly contributed an obstacle to the progress of medical science. Our creed that every therapeutic discovery made by a reputable member of the profession must forthwith become public property is undoubtedly a wise one, and we are far from deprecating its observance. At the same time it cannot be denied that the embargo we have laid upon the commercial exploitation of such discoveries does obstruct the furtherance of research. This is not to say that there is any lack of men ready to devote themselves to this mission, for indeed there are plenty, but they perform their labours for the most part under grave disadvantages. We refer, of course, to the lack of money. Wherever, apart from medicine, a branch of science is of public utility it is capable of commercial exploitation, and far-seeing business men do not hesitate to exploit it. Being satisfied that there is "money in it," as the saying goes, they put money into it. They engage for the prosecution of the researches required the best brains they can find among those who have made the subject their own, and place their workers in the most favourable position for achieving the anticipated result. In consequence the utilitarian sciences, as we may call them, have advanced with amazing rapidity; witness particularly the commercial applications of chemistry and the physical sciences. The science of medicine, on the other hand, though in itself utilitarian to the topmost degree, in consequence of our self-denying ordinance stands in the catalogue of academic sciences, such as palæontology, which have no meretricious attractions; and, like its fellows in the catalogue, the science of medicine has always experienced the disabilities associated with an empty purse. If, in spite of this misfortune, we can still point with pride to a rich harvest of achievement, we but emphasise the extraordinary magnetism of a subject which offers its worshippers so trifling a return in hard cash.

But although it is comforting to feel that experimental medicine in its varied branches can on its own merits command a host of zealous disciples prepared to work for a pittance or even at their own charges, it will hardly be disputed that if the way of the researcher were made more smooth he could cover more ground than that over which he now struggles often under conditions of considerable personal hardship. The present system is extremely wasteful of our best intelligences in this department. Take for example the case of bacteriological research. A medical student develops an inclination for bacteriology. He cultivates his hobby during the few idle moments of his student career, and pursues it in its practical applications while he is a house-physician or house-surgeon. He next perhaps obtains a research scholarship which enables him to devote his whole time for a year or two to the subject of his choice. In due time, having thoroughly grounded himself in the elements—in itself an immense task when bacteriology is concerned—he becomes a demonstrator of pathology at his hospital school. Here he immediately becomes immersed by the sea of routine, and realises, moreover, that the demonstration of pathology is not a lucrative business. The greater part of his day is spent in the drudgery of a pathological department, the search for tubercle bacilli in sputa, the identification of diphtheria bacilli in swabs from throats. Elementary classes also claim his attention, and, since the establishment finds it cheaper to engage a highly qualified medical man than to pay for an additional laboratory boy to cut sections for class purposes, he probably finds himself occupied for many hours every week in this highly intellectual pursuit. With advancing seniority he is, of course, able to free himself from the most mechanical part of the laboratory routine, but the classes have probably been replaced by formal lectures whose preparation makes large drafts upon his time. Such lecturing, together with administrative duties and hack-work undertaken to swell his lean exchequer, form a constantly recurring obstacle to the full fruition of such genius for original work as he may happen to possess.

It is surely a great pity that matters should stand in this unsatisfactory position. The pathological departments of our great hospitals have at hand a supply of material for research which is practically inexhaustible. It is not crying for the moon to say that those who have worked their way to the headship of these departments should be enabled to use their opportunities to the best advantage. Something has been done in this direction by the endowment of special research laboratories; but these, valuable as they are, must always be denied the ready access to clinical material which is offered so lavishly to the pathological departments of large hospitals. If once the charitable public could be made to realise the enormous benefits likely to accrue to them indirectly by the proper endowment of hospital pathology, the waste of talent and opportunity to which we have referred would soon be a thing of the past. Unfortunately the slow labours of the laboratory bench do not readily touch the popular imagination, while the wide diffusion of appeals to prejudice in the shape of anti-vivisection literature have undoubtedly deflected sympathy from a branch of philanthropic work which has few equals in merit and no superiors. What we have to do is to disseminate a knowledge of the true facts of the case and appeal to the reason of our audience. Unhappily purse-strings open far more quickly at the call of emotion than of reason; and, as we have seen, pathology seems to stir no emotions but unworthy ones, since the public knows little of it beyond what they hear from its fanatical opponents. It is therefore high time for an official propaganda designed to combat the one-sided arguments with which the lay world is continually plied. The newly-formed Research Defence Society, designed for this worthy mission, has had a magnificent welcome, in which we feel sure that all moderate people will join.

